# Exploring patterns in mental health treatment and interests of single adults in the United States: a secondary data analysis

**DOI:** 10.3389/fpubh.2024.1292603

**Published:** 2024-04-22

**Authors:** Amanda N. Gesselman, Ellen M. Kaufman, Lee Y. S. Weeks, Zoe Moscovici, Margaret Bennett-Brown, Olivia R. Adams, Jessica T. Campbell, Malia Piazza, Lucy Bhuyan, Simon Dubé, Jessica J. Hille, Justin R. Garcia

**Affiliations:** ^1^Kinsey Institute, Indiana University Bloomington, Bloomington, IN, United States; ^2^Department of Gender Studies, College of Arts and Sciences, Indiana University Bloomington, Bloomington, IN, United States; ^3^Communication Studies, College of Media and Communications, Texas Tech University, Lubbock, TX, United States; ^4^Department of Gender, Sexuality, and Women’s Studies, College of Liberal Arts and Sciences, University of Florida, Gainesville, FL, United States; ^5^Department of Anthropology, College of Arts and Sciences, Indiana University Bloomington, Bloomington, IN, United States; ^6^Department of Counseling and Educational Psychology, School of Education, Indiana University Bloomington, Bloomington, IN, United States; ^7^Department of Psychology, Faculty of Arts and Science, Concordia University, Montreal, QC, Canada

**Keywords:** mental health treatment-seeking, client characteristics, gender identity, ethnic/racial identity, psychotherapy

## Abstract

**Objective:**

The objective of this study is to examine mental health treatment utilization and interest among the large and growing demographic of single adults in the United States, who face unique societal stressors and pressures that may contribute to their heightened need for mental healthcare.

**Method:**

We analyzed data from 3,453 single adults, focusing on those with possible mental health treatment needs by excluding those with positive self-assessments. We assessed prevalence and sociodemographic correlates of mental health treatment, including psychotherapy and psychiatric medication use, and interest in attending psychotherapy among participants who had never attended.

**Results:**

26% were in mental health treatment; 17% were attending psychotherapy, 16% were taking psychiatric medications, and 7% were doing both. Further, 64% had never attended psychotherapy, of which 35% expressed interest in future attendance. There were differences in current psychotherapy attendance and psychiatric medication use by gender and sexual orientation, with women and gay/lesbian individuals more likely to engage in both forms of mental health treatment. Additionally, interest in future psychotherapy among those who had never attended varied significantly by age, gender, and race. Younger individuals, women, and Black/African-American participants showed higher likelihoods of interest in psychotherapy.

**Conclusion:**

Our research highlights a critical gap in mental health treatment utilization among single adults who may be experiencing a need for those services. Despite a seemingly higher likelihood of engagement in mental health treatment compared to the general population, only a minority of single adults in our sample were utilizing mental health treatment. This underutilization and the observed demographic disparities in mental health treatment underscore the need for targeted outreach, personalized treatment plans, enhanced provider training, and policy advocacy to ensure equitable access to mental healthcare for single adults across sociodemographic backgrounds.

## Introduction

In recent years, the demographic landscape of the United States has witnessed a significant shift towards increased rates of singlehood (i.e., being romantically unpartnered), with a growing proportion of adults choosing to remain single or experiencing single life due to various circumstances (1–5). As a group, single adults face distinct social, psychological, and economic challenges, compared to romantically partnered adults. Single adults often navigate a complex social environment that prioritizes romantic relationships and their resulting familial relationships as indicators of personal success and happiness (6–8). As a result, single adults face societal stigma and pressures that can contribute to feelings of isolation, loneliness, and perceived inadequacy (9, 10). Further, singlehood introduces a set of unique stressors and life situations that may not be as prevalent among partnered individuals. These include making financial and living arrangements independently, providing extensive care to ill or aging family members, and managing one’s own physical health within a healthcare system that restricts in-person support to individuals who are legally or biologically related (4, 11). Particularly among those who are involuntarily single, singlehood may also prevent fulfillment of certain personal goals throughout the lifecycle (e.g., parenthood; long-term romantic companionship), exacerbating the negative mental health impact produced by other individual and societal factors unique to this population (12, 13). Taken together, these experiences could lead to a heightened need for mental health treatment among single adults.

Social causation theory (14, 15) offers a valuable lens through which to understand the increased need for mental health treatment among single adults. This theory suggests that social factors cause or worsen mental health issues. For example, investigations informed by this theory have shown that the stress and environmental adversity associated with having a low socioeconomic status contribute to higher probabilities for developing anxiety, depression, or other psychiatric disorders (16, 17). Reframed in the context of single adults, social causation theory might suggest that factors such as the presence or absence of supportive social relationships, societal expectations and pressures, and stigma associated with being single could evoke or exacerbate mental health issues. Considering that one-third of the adult population in the United States is currently single and that this population appears to be growing (2), it is important to understand how single adults utilize mental health treatment resources. Examining current uptake within single adults, and pinpointing demographic subgroups of single adults less likely to seek out resources when in need, is crucial for understanding how social structures and societal norms impact individual well-being.

Mental health disorders (e.g., anxiety, depression) affect millions worldwide, with rates rising in recent years, and further intensified by the COVID-19 pandemic (18–22). Mental health issues can severely impact personal, economic, and social well-being [e.g., (23–25)], which are known to further exacerbate existing mental health difficulties (26). In the U.S., mental health treatment primarily consists of psychotherapy and/or prescribed psychiatric medications (27, 28). Psychotherapy involves talking with a trained therapist, with techniques ranging from cognitive-behavioral to psychodynamic approaches (29, 30). Psychiatric medications, like antidepressants and anxiolytics, alter neurotransmitter activity to address mental health issues (31, 32). Despite the need for mental health treatment, reports suggest that treatment may be underutilized (33–38).

Largescale reports of mental health treatment utilization in the United States are sparse. However, the U.S. Centers for Disease Control (CDC) reports that around one in five American adults receive mental health treatment annually, with prescription psychiatric medication use more common than psychotherapy (32). According to their study, 17% of U.S. adults were receiving mental health treatment in the form of prescription psychiatric medication while 10% of U.S. adults were receiving psychotherapy/counseling (32). It should be noted that Terlizzi and Norris’ (32) study did not account for participants’ need for mental health treatment. Instead, results are derived from a nationally representative household survey of adults in the U.S., which may have included many adults without a need for these services. As such, it is difficult to accurately estimate the percentage of U.S. adults who should be seeking mental health treatment. However, studies conducted in other countries suggest that adults in the U.S. are underutilizing mental healthcare. While mental health treatment utilization was between 10 and 17% for U.S. adults (32), other countries reported that between 32 and 71% of the adult population had accessed mental health treatment (39–41). Further national studies are crucial to understand U.S. adults’ need for mental health treatment and pursuit of those services.

In the current study, we focus on single adults with self-reported suboptimal mental health—excluding those with higher self-assessments—to investigate their uptake or interest in mental health treatment. Singles are a heterogeneous group, however; to avoid viewing this group monolithically, it is important to investigate how demographic factors aligning with different identities and/or lived experiences contribute to their uptake and interest in mental health treatment. In particular, research conducted in the U.S. and elsewhere has shown that factors like gender, age, socioeconomic status, and race influence mental health treatment-seeking behavior. Women are more likely to be receiving mental health treatment compared to men (32, 42–44). This gender effect between men and women has been found consistently across sampled countries, but no further investigations into gender identity and mental health treatment-seeking are found in the literature. In particular, while gender identity is clearly impactful on treatment-seeking, only studies conducted with adolescent participants have examined differences in mental health treatment as a function of transgender or cisgender identities (45). We do know, however, that transgender and genderqueer individuals are exposed to more societal and structural risks that increase their need for mental health services (46). This disparity in exposure to risks is a reflection of “minority stress”—high levels of stress endured by members of stigmatized minority groups—a factor that exacerbates challenges to mental health for many people, including those with minoritized gender, racial/ethnic, and sexual orientation identities (47–50).

With regards to age, most studies have found that younger people are more likely than older people to be in psychotherapy (32, 43, 44), although Terlizzi and Norris’ (32) sample of U.S. adults found a reversed pattern of results for psychiatric medication use—older age was associated with more use. Further, there appears to be a robust effect for socioeconomic standing, but with a different direction of effect depending on country and type of mental health treatment assessed. In Brazil and Finland, higher education level and occupational status were associated with seeking mental health treatment [(42, 43); but see Suokas et al. (51)]. Conversely, in Spain, people with lower education and income levels were more likely to seek mental health treatment (44). There are no known studies examining the association between income and mental health treatment utilization in a U.S. sample. However, in line with social causation theory, lower income levels are associated with higher prevalence of mental illness in the U.S. (52). Reflecting the systemic bias inherent in the U.S.’s societal hierarchy, people with minoritized identities or demographic backgrounds often have the lowest income (53). Taken together, these findings would suggest that people engaged in mental health treatment are more likely to have lower income and to hold a minority identity. However, in their CDC study, Terlizzi and Norris (32) found that people who identified their race/ethnicity as non-Hispanic white—a majoritized identity—were more likely to have received mental health treatment than Hispanic, non-Hispanic Black, and non-Hispanic Asian adults. Further research is needed to understand how mental health treatment uptake and interest varies by income and race in U.S adults.

Amassed, the small existing literature suggests that within a population of single adults who may need mental health treatment, mental health treatment uptake and interest is more likely for people who identify as women (vs. men); younger in age (vs. older); potentially white, non-Hispanic (vs. other races/ethnicities); and potentially higher (vs. lower) in income. In addition to these, there are a few key demographic factors that have not yet been investigated but may drive differences in mental health treatment utilization and interest. For example, research indicates that non-heterosexual (e.g., gay or lesbian, queer) individuals are exposed to a multitude of societal and structural risks that predispose them to a greater need for mental health services (54–56), yet there’s insufficient exploration into how these pressures translate into actual mental health treatment engagement compared to heterosexual individuals. Further, because this investigation takes place in the single adult population, it is important to assess the impact of single parenthood on mental health treatment. In addition to increased stress, parenthood evokes hormonal changes, interpersonal stress, and sleep disturbances that may subsequently challenge mental well-being (57–61). Single parents might experience these challenges more acutely than partnered parents, as a result of the added responsibilities and limited support (62, 63). In the current study, we investigate differences in mental health treatment uptake and interest by age, gender, income, and race, as prior studies have done. Further, we undertake the first known investigation into differences in mental health treatment uptake and interest by sexual orientation and parenthood.

In the current study, we conducted a secondary data analysis on an existing national dataset of single adults in the United States. Selecting only those who self-rated their mental health over the last year as “poor,” “fair,” or “good”—and omitting those who self-rated their mental health as “very good” or “excellent”—we examined engagement and interest in mental health treatment among 3,453 single adults to answer the following research questions. Among single adults who may need mental health treatment:

1. How many were undergoing mental health treatment at time of survey? We examined mental health treatment in the form of attending psychotherapy/counseling, using psychiatric medications, or both.
2. What sociodemographic characteristics of singles are associated with undergoing mental health treatment (i.e., psychotherapy/counseling, psychiatric medication use, or both)?
3. Of those who had never attended psychotherapy, how many were interested in attending psychotherapy in the future, and what sociodemographic characteristics are most associated with interest?

## Methods

Below we report how we determined our sample size, all data exclusions, and all measures used in the current study. This study did not employ manipulations.

### Participants

The initial sample included 5,001 single adults. We restricted the sample to only those who reported their mental health as “poor,” “fair,” or “good” on a 5-point Likert scale ranging from poor (1) to excellent (5). Participants who rated their mental health as “very good” (*n* = 1,012) or “excellent” (*n* = 536) were removed from the sample. We report descriptive statistics below for mental health treatment utilization and interest among a sample of 3,453 single adults (see Table 1 for demographic distributions). However, further sample restrictions were required before we could investigate comparisons by gender, sexual orientation, or race/ethnicity, resulting in an analytic sample of 2,902 participants; see the Data Analysis Plan section below for details.

**Table 1:** Demographic distributions for the sample of participants who may need mental health treatment.

Variables	*N* = 3,453
Age	*M* = 42.63 years, *SD* = 17.47
Gender	
Man	1,276 (37.0%)
Woman	2,130 (61.0%)
Non-binary (e.g., genderqueer, genderfluid, agender)	38 (1.1%)
Another identity not listed	4 (0.1%)
Do not know	2 (0.1%)
Choose not to answer	3 (0.1%)
Transgender identity	
Transgender	101 (2.9%)
Not transgender	3,204 (92.8%)
Unsure	92 (2.7%)
Choose not to answer	56 (1.6%)
Ethnicity/race	
Black/African American	590 (17.1%)
East Asian	104 (3.0%)
Hispanic/Latino	264 (7.6%)
Native American/Alaskan Native	26 (0.8%)
South Asian	33 (1.0%)
White/Caucasian	2,150 (62.3%)
Another identity not listed	20 (0.6%)
Two or more races/ethnicities	266 (7.7%)
Sexual orientation	
Straight/heterosexual	2,907 (84.2%)
Gay or Lesbian	186 (5.4%)
Bisexual	249 (7.2%)
Pansexual	45 (1.3%)
Asexual	48 (1.4%)
Another identity not listed	18 (0.5%)
Household income	
Less than $15,000	681 (19.7%)
$15,000–$29,999	835 (24.2%)
$30,000–$44,999	635 (18.4%)
$45,000–$59,999	476 (13.8%)
$60,000–$74,999	305 (8.8%)
$75,000–$99,999	278 (8.1%)
$100,000–$149,000	187 (5.4%)
$150,000 or more	56 (1.6%)
Parental status	
Has children	1,369 (39.6%)
Does not have children	2,084 (60.4%)

### Procedure

Data were collected as part of the annual *Singles in America* (SIA) study. The current study was conducted as a secondary data analysis of de-identified market research data collected by an outside party. The authors of this manuscript have not interacted with participants and have access only to a de-identified dataset. Based on the nature of these secondary analyses of anonymized data, the current research is exempt from the federal regulations at 45 CFR part 46 (64). Participants must have been at least 18 years old, fluent in English, and single (i.e., romantically unpartnered). Singlehood was defined for participants as being unmarried and not in a committed romantic relationship. There were no requirements for the length of singlehood. Participants were recruited by Dynata (Dallas, TX, United States), using independent opt-in Internet research panels for quota-based cross-sectional surveying. Panelists were drawn from a diverse pool of participants who have been recruited over several years from many venues, including paper and electronic mailings and internet recruitment. Recruitment was balanced in real-time so that demographic distributions (i.e., age, gender, ethnicity, region, income) in the sample were closely aligned with demographic distributions published in the most recent Current Population Survey conducted by the U.S. Bureau of the Census.

Participants received a recruitment message from Dynata inviting participation for financial remuneration (~$5 USD). Panelists were screened to ensure survey engagement, with those straight-lining responses or moving too quickly through panels removed. Participants completed the full survey; there is no missing data due to participants opting to skip questions. All data were collected over the Internet. SIA is sponsored by the relationship company Match; however, participants were not recruited or drawn from the Match population or subsidiary sites. No *a priori* power analyses were conducted; the Singles in America survey aims to collect a sample of 5,000 respondents yearly. This study was not pre-registered.

### Measures

#### Demographics

Participants reported their age, gender and transgender identity, income, parenting status, sexual orientation (straight/heterosexual; gay/lesbian; bisexual; other), and race/ethnicity (White; Black/African-American; Hispanic/Latino; South Asian; East Asian; North American Indian/Alaska Native/Pacific Islander; other). Measures of gender were based on Haupert et al. (65) for inclusive measurement. First, participants selected gender identity from this list: *man, woman, non-binary (e.g., genderqueer, genderfluid), agender, another identity not listed, do not know, choose not to answer*. Next, they answered, “‘Transgender’ describes people whose gender identity or expression is different, at least part of the time, from the sex assigned to them at birth. Do you consider yourself to be transgender?” with one of the following options: *yes*, *no*, *do not know*, and *choose not to answer*. Regarding race/ethnicity, note that participants were allowed to select all identities that applied; however, for analytic purposes, we recoded participants who identified with multiple racial/ethnic identities into their own category.

#### Self-reported mental health

Participants responded to the following question, “How would you rate your average mental health over the last year?” Responses were, “poor” (1), “fair” (2), “good” (3), “very good” (4), and “excellent” (5). Note that this question was used to restrict the final analytic sample, such that participants who selected “very good” or “excellent” were removed.

#### Mental health treatment

Participants responded to a question assessing broader medication use: “Are you currently taking/using any of the following? Select all that apply.” The list included 15 options (e.g., hormonal birth control, testosterone, prescription painkillers). Of note, the list included four options relevant to mental health treatment. These were, “SSRI (e.g., Lexapro, Prozac, Zoloft)”; “SNRI (e.g., Pristiq, Cymbalta, Effexor)”; “MAOI (e.g., Marplan, Nardil)”; and “Wellbutrin.”

Participants also responded to: “Have you been in therapy, counseling, or some form of mental healthcare in the last 3 years?” Response options were *Yes, currently*; *Yes, in the past but not now*; *No, but I’m interested in seeking therapy/treatment*; and *No, and I’m not interested in seeking therapy/treatment.*

### Data analysis plan

Within the sample of participants who may need mental health treatment (i.e., who rated their mental health as poor, fair, or good), we first examined descriptive statistics for demographics and mental health treatment variables. Next, we conducted three binary logistic regressions. However, due to cell size restrictions, a number of adjustments were made prior to regression modeling. First, we recoded the gender and transgender identity variables. People who did not identify as a man or woman (*n* = 47), or did not select “yes” or “no” for the transgender identity question (*n* = 138), were removed from the analytic sample. Second, we restricted the analyses to compare participants who identified their race/ethnicity as white, Black/African-American, or Hispanic/Latino. Due to small cell sizes—and particularly due to low engagement with either form of mental health treatment or interest in psychotherapy—we could not analytically compare mental health treatment engagement or interest for East or South Asian participants (*n* = 33 and 104, respectively), Native American or Alaskan Native participants (*n* = 26), participants who identified as another race or ethnicity not listed (*n* = 20), or participants who identified as two or more races/ethnicities (*n* = 266). Likewise, we could not compare mental health treatment or interest for people identifying their sexual orientation as pansexual (*n* = 45), asexual (*n* = 48), or another orientation not listed (*n* = 18); instead, we compare participants identifying their sexual orientation as heterosexual, gay/lesbian, and bisexual. Finally, we did not have adequate statistical power to compare transgender and cisgender participants, as only 101 participants identified as transgender. However, we did not remove these individuals from the sample unless they specified their gender as being an identity other than man or woman.

The final analytic sample for demographic comparisons in regression models was 2,902 single adults. In the regression models, the dependent variables were whether participants were attending psychotherapy (model 1), whether they were taking psychiatric medications (model 2), and among participants who had not been in psychotherapy, whether they were interested in psychotherapy in the future (model 3; all 0 = no, 1 = yes). Predictor variables were: age (mean-centered); income; gender (0 = men, 1 = women; in regression models, men served as the comparison group); two sexual orientation dummy codes (0 = heterosexual, 1 = gay/lesbian, bisexual; in regression models, heterosexual served as the comparison group); parent status (0 = does not have children, 1 = has children); and two dummy codes for race/ethnicity (0 = White, 1 = Black/African-American, Hispanic/Latino, White served as comparison). Because these analyses include multiple comparisons of the same individuals, we implemented a Bonferroni correction. Only effects reaching significance at *p* ≤ 0.001 are interpreted below. All data and analytic codes can be found at https://osf.io/zh3kd/?view_only=725060e1f96546f5ad5acdf9c5881aab.

## Results

### Prevalence of singles in mental health treatment

Within the sample of participants who may need mental health treatment, 26.2% (*n* = 902) of the sample were in mental health treatment at the time of the survey (i.e., psychotherapy or psychiatric medication use): 16.5% were in psychotherapy, 16.3% were taking psychiatric medications, and 6.7% were doing both. Additionally, 19.1% had attended psychotherapy previously but not currently. In total, 35.6% of the sample had experience with psychotherapy; 64.4% had not been in psychotherapy before, of which 35.4% were interested in attending psychotherapy in the future.

### Sociodemographic associations with current mental health treatment

We examined associations between age, income, gender, race, sexual orientation, and parental status with attending psychotherapy, taking psychiatric medications, and interest in pursuing psychotherapy. We report percentages of engagement and interest in mental health treatment by demographics in Table 2 (psychotherapy attendance) and Table 3 (psychiatric medication use). Regression coefficients can be found in Tables 4–6.

**Table 2:** Sample demographics by therapy attendance and interest in pursuing therapy.

	Has received therapy (*n* = 1,138)		Has not received therapy (*n* = 2,076)	
Variables	Currently (*n* = 526)	In the past (*n* = 612)	Interested in therapy (*n* = 718)	Uninterested in therapy (*n* = 1,358)
Age	*M* = 42.53 years, *SD* = 15.39 years	*M* = 38.06 years, *SD* = 15.53 years	*M* = 37.85 years, *SD* = 15.13 years	*M* = 49.00 years, *SD* = 18.31 years
Gender				
Man	161 (30.6%)	233 (38.1%)	251 (35.0%)	553 (40.7%)
Woman	365 (69.4%)	379 (61.9%)	467 (65.0%)	805 (59.3%)
Transgender status				
Transgender	14 (2.7%)	26 (4.2%)	22 (3.1%)	17 (1.3%)
Not transgender	512 (97.3%)	586 (95.8%)	696 (96.9%)	1,341 (98.7%)
Ethnicity/race				
Black/African American	93 (17.7%)	104 (17.0%)	166 (23.1%)	183 (13.5%)
East/South Asian	10 (1.9%)	23 (3.8%)	31 (4.3%)	66 (4.9%)
Hispanic/Latino	30 (5.7%)	57 (9.3%)	78 (10.9%)	76 (5.6%)
White/Caucasian	356 (67.7%)	365 (59.6%)	381 (53.1%)	950 (70.0%)
Two or more races/ethnicities	37 (7.0%)	63 (10.3%)	62 (8.6%)	83 (6.1%)
Sexual orientation				
Straight/heterosexual	415 (78.9%)	509 (83.2%)	594 (82.7%)	1,243 (91.5%)
Gay or lesbian	45 (8.6%)	30 (4.9%)	41 (5.7%)	55 (4.1%)
Bisexual	49 (9.3%)	56 (9.2%)	61 (8.5%)	48 (3.5%)
Pansexual	8 (1.5%)	7 (1.1%)	12 (1.7%)	3 (0.2%)
Asexual	9 (1.7%)	10 (1.6%)	10 (1.4%)	9 (0.7%)
Household income				
Less than $15,000	136 (25.9%)	105 (17.2%)	137 (19.1%)	228 (16.8%)
$15,000–$29,999	135 (25.7%)	145 (23.7%)	163 (22.7%)	345 (25.4%)
$30,000–$44,999	78 (14.8%)	111 (18.1%)	145 (20.2%)	262 (19.3%)
$45,000–$59,999	68 (12.9%)	88 (14.4%)	106 (14.8%)	186 (13.7%)
$60,000–$74,999	40 (7.6%)	51 (8.3%)	71 (9.9%)	128 (9.4%)
$75,000–$99,999	34 (6.5%)	54 (8.8%)	59 (8.2%)	111 (8.2%)
$100,000–$149,000	30 (5.7%)	48 (7.8%)	30 (4.2%)	68 (5.0%)
$150,000 or more	5 (1.0%)	10 (1.6%)	7 (1.0%)	30 (2.2%)
Parental status				
Does not have children	317 (60.3%)	376 (61.4%)	452 (63.0%)	776 (57.1%)
Has children	209 (39.7%)	236 (38.6%)	266 (37.0%)	582 (42.9%)

**Table 3:** Sample demographics for participants who were and were not taking psychiatric medications.

Variables	Taking psychotropic medications (*n* = 516)	Not taking psychotropic medications (*n* = 2,698)
Age	*M* = 43.10 years, *SD* = 16.11 years	*M* = 43.41 years, *SD* = 17.64 years
Gender		
Man	135 (26.2%)	1,063 (39.4%)
Woman	381 (73.8%)	1,635 (60.6%)
Transgender status		
Transgender	26 (5.0%)	53 (2.0%)
Cisgender	490 (95.0%)	2,645 (98.0%)
Ethnicity/race		
Black/African American	60 (11.6%)	486 (18.0%)
East/South Asian	7 (1.4%)	123 (4.6%)
Hispanic/Latino	25 (4.8%)	216 (8.0%)
White	384 (74.4%)	1,668 (61.8%)
Two or more races/ethnicities	40 (7.8%)	205 (7.6%)
Sexual orientation		
Straight/heterosexual	394 (76.4%)	2,367 (87.7%)
Gay or lesbian	50 (9.7%)	121 (4.5%)
Bisexual	50 (9.7%)	164 (6.1%)
Pansexual	11 (2.1%)	19 (0.7%)
Asexual	11 (2.1%)	27 (1.0%)
Household income		
Less than $15,000	113 (21.9%)	493 (18.3%)
$15,000–$29,999	124 (24.0%)	664 (24.6%)
$30,000–$44,999	80 (15.5%)	516 (19.1%)
$45,000–$59,999	76 (14.7%)	372 (13.8%)
$60,000–$74,999	52 (10.1%)	238 (8.8%)
$75,000–$99,999	36 (7.0%)	222 (8.2%)
$100,000–$149,000	29 (5.6%)	147 (5.4%)
$150,000 or more	6 (1.2%)	46 (1.7%)
Parental status		
Does not have children	309 (59.9%)	1,612 (59.7%)
Has children	207 (40.1%)	1,086 (40.3%)

**Table 4:** Demographic characteristics predicting current psychotherapy attendance.

Variable	OR	95% CI	*p*-value
Age	1.00	0.99–1.00	0.120
Income	0.93	0.88–0.98	0.011
Gender^a^	1.48	1.19 – 1.85	< 0.001
Sexual orientation: gay/lesbian^b^	2.36	1.62–3.45	< 0.001
Sexual orientation: bisexual	1.36	0.93–1.98	0.108
Parental status	0.92	0.74–1.15	0.455
Race/ethnicity: Black/African-American^c^	0.99	0.77–1.28	0.934
Race/ethnicity: Hispanic or Latino	0.55	0.36–0.84	0.005

a Men served as the reference group.

b The sexual orientation category “Straight/heterosexual” served as the reference group.

c White/Caucasian served as the reference group for race/ethnicity.

**Table 5:** Demographic characteristics predicting psychiatric medication use.

Variable	OR	95% CI	*p*-value
Age	0.99	0.98–1.00	0.005
Income	0.97	0.92–1.03	0.271
Gender^a^	2.05	1.62–2.60	<0.001
Sexual orientation: Gay/lesbian^b^	2.87	1.97–4.20	<0.001
Sexual orientation: Bisexual	1.46	1.01–2.12	0.043
Parental status	0.98	0.78–1.23	0.880
Race/ethnicity: Black/African-American^c^	0.51	0.38–0.68	<0.001
Race/ethnicity: Hispanic or Latino	0.37	0.23–0.58	<0.001

a Men served as the reference group.

b The sexual orientation category “Straight/heterosexual” served as the reference group.

c White/Caucasian served as the reference group for race/ethnicity.

**Table 6:** Demographic characteristics predicting interest in future psychotherapy attendance, among those who had never attended psychotherapy.

Variable	OR	95% CI	*p*-value
Age	0.96	0.96–0.97	< 0.001
Income	1.01	0.95–1.07	0.842
Gender^a^	1.54	1.24–1.92	< 0.001
Sexual orientation: Gay/lesbian^b^	1.58	0.96–2.57	0.069
Sexual orientation: Bisexual	1.52	0.98–2.35	0.060
Parental status	1.15	0.91–1.44	0.238
Race/ethnicity: Black/African-American^c^	1.56	1.21–2.01	< 0.001
Race/ethnicity: Hispanic or Latino	1.39	0.98–1.98	0.067

a Men served as the reference group.

b The sexual orientation category “Straight/heterosexual” served as the reference group.

c White/Caucasian served as the reference group for race/ethnicity.

#### Current psychotherapy attendance

In the regression model, there were significant differences in current psychotherapy attendance by gender and sexual orientation. Results suggest that women are more likely than men, and gay/lesbian individuals are more likely than heterosexual individuals, to be currently engaged in psychotherapy. Age, income, parenthood, and race/ethnicity were not significant predictors of psychotherapy attendance at *p* ≤ 0.001. See Table 4 for regression coefficients.

#### Current psychiatric medication use

There were significant differences in the odds of currently using psychiatric medication use by gender, sexual orientation, and race. Results suggest that women are more likely than men, gay/lesbian individuals are more likely than heterosexual individuals, and both Black/African-American and Hispanic/Latino individuals are more likely than white individuals to be currently taking psychiatric medications. Age, income, and parenthood were not significant predictors of psychotherapy attendance at *p* ≤ 0.001. See Table 5 for regression coefficients.

#### Interest in attending psychotherapy in the future

Among the subgroup of participants who had never attended psychotherapy, there were significant differences in the odds of being interested in future psychotherapy attendance by age, gender, and race. Results suggest that younger participants are more likely than older participants, women are more likely than men, and Black/African-American participants are more likely than white participants to be interested in attending psychotherapy in the future. Income, sexual orientation, and parenthood were not significant predictors of psychotherapy attendance at *p* ≤ 0.001. See Table 6 for regression coefficients.

## Discussion

Single adults face a variety of unique societal stressors, including social stigma, a lack of systemic support, and increased financial and social obligations compared to partnered peers (4). These experiences may produce negative mental health outcomes throughout the lifespan, subsequently producing a need for mental health treatment (9, 10). In our study, we estimated mental health treatment rates among single American adults, who comprise around one-third of the adult population (2, 66–68). Among those who self-reported their mental health as suboptimal, we identified demographic subgroups who have higher likelihoods of utilizing mental health treatment resources and who may be more receptive to mental health treatment in the future. Our findings shed light on the extent to which single adults in the U.S. are currently engaging in mental health treatment and helps to pinpoint areas of need based on demographic background.

At the time of the survey, in August 2022, more than one-quarter (26%) of single adults in our sample were using mental health resources. In particular, 17% were currently engaged in psychotherapy and 16% were taking psychiatric medications (SSRIs, SNRIs, MAOIs, or Wellbutrin). The prevalence of mental health treatment in this sample of single adults was more substantial than reports generated from the general population [i.e., 10% in psychotherapy, 17% using psychiatric medications; (32)]. This suggests that single adults may seek out mental health treatment to a greater extent than the average population, and perhaps more than romantically partnered individuals. Future research should investigate this comparison.

With regards to demographic differences, we found that women were more likely than men to receive or be interested in mental health treatment. Further, we found that age was unassociated with current psychotherapy attendance or psychiatric medication use, but younger individuals were more likely to be interested in attending psychotherapy in the future, compared to older individuals. Gendered risk factors, societal influences, stigma around male help-seeking, and normalization of mental health conversations among younger audiences on social media platforms could contribute to these patterns (69–72). The COVID-19 pandemic has also had a profound impact on mental health and well-being across populations; younger individuals with less life experience may be less equipped to adapt to pandemic stressors and thus more interested in professional mental health treatment (73).

We found racial and ethnic differences in mental health treatment and interest. Although there were no racial/ethnic differences in the likelihood of current psychotherapy attendance, Black/African-American and Hispanic/Latino singles were less likely than White singles to report psychiatric medication use. There is no prior empirical literature to explain these differences. However, because psychiatric medications are procured through visits with a healthcare provider with the ability to prescribe medications, people who have experienced discrimination in healthcare settings may be less inclined to pursue mental health treatment via this route (74–77). Of note, Black/African-American participants were more likely than white participants to be interested in attending psychotherapy in the future. This suggests a nuanced understanding of mental health treatment preferences among racial and ethnic groups, highlighting the importance of considering historical and systemic factors that influence these preferences. It underscores the need for culturally sensitive approaches in mental health treatment provision, which can address and mitigate barriers to accessing treatment, particularly in the context of psychiatric medication.

In terms of sexual orientation, gay and lesbian participants were more likely to be currently engaged in psychotherapy or using psychiatric medications than were heterosexual participants. These findings align with prior work showing that people identifying as a sexual minority are more likely to seek out mental health treatment [e.g., (78, 79)]. Further, these findings demonstrate the impact of minority stress on the need for mental health treatment (55): gay and lesbian participants likely experience a number of stressors related to their around their sexual identity and minoritized societal standing, which could increase their need for mental health services compared to heterosexual peers.

Finally, we did not find any differences in mental health treatment utilization or interest with respect to parenthood status or income. Although the transition to parenthood can bring about immense stress (57–61), and single parenthood may exacerbate that stress (e.g., 63), we did not observe differences in current mental health treatment uptake or interest as a function of whether or not participants had children. Our dataset did not account for the age of children or whether participants had full custody of their children, which would dictate whether the child(ren) resided with the participant. These factors have the potential to create substantial differences in the stress resulting from parenting, as well as in the amount of time one has to pursue treatment. Future research should investigate these discrepancies.

Prior literature suggests that lower income would be associated with greater mental health treatment uptake (52, 53), but income was not a meaningful factor in our study. Mental health treatment is often privatized and potentially costly in the U.S. (80). However, lower incomes have been linked to greater use of mental health resources offered as free services (81, 82). Further, one in five U.S. adults are enrolled in the Medicaid program for people with low income (83), which offers some financial coverage for a range of behavioral health conditions [e.g., substance use disorder; Medicaid.gov (84)]. Taken together, our lack of significant results for income may be explained by the ability of higher-income participants to afford treatment, and the accessibility of mental health treatment for lower-income participants. Future research is needed to understand how socioeconomic status dictates mental health treatment utilization and interest.

### Limitations

The current research has several limitations. First, it relies on self-report data which requires participants to be honest and open. As the survey dealt with the stigmatized topic of mental health treatment, some participants may have answered in a manner more socially desirable than truthful [e.g., (85)]. Relatedly, online survey research studies must acknowledge a self-selection bias, in which participants who are more interested in a topic may be more likely to take the survey. This bias violates probability theory; therefore online quota-based samples like the one used in the current study cannot be considered a random sample (86). This limits generalizability of our findings. Future research should employ random sampling techniques to help reduce self-selection bias. Future researchers could also cross-verify self-reported data about engagement in mental health treatment with healthcare records, to ensure that participants are accurately reporting their mental health treatment uptake.

This study is also limited by the ways in which the survey assessed psychotherapy attendance and psychiatric medication use. There was a lack of specificity regarding types of psychotherapy in which participants were engaged or interested. We assumed that participants understood the term “therapy/counseling” to mean sessions with a licensed mental health professional. However, participants might have considered alternative forms of care as psychotherapy, which would not be recognized as psychotherapy by the academic/scientific community. Future research should assess the specific types of therapeutic support that are sought after and preferred. Additionally, the survey did not include the full range of available psychiatric medications or assess interest in future use of such psychiatric medications. Consequently, the study may have unintentionally excluded participants using other types of psychiatric medications, providing an incomplete view of psychiatric medication use among singles. In future studies, it is essential to include all available forms of psychiatric medication to prevent unintentional exclusions and possible oversights. Relatedly, the survey may inadequately represent individuals with severe mental illness. There is often a co-occurrence of poverty and severe mental illness, including homelessness and joblessness (87). People with severe mental illness may be underrepresented in this research due to barriers to accessing online surveys.

After restricting our analytic sample to only participants who self-rated their mental health in the past year as suboptimal (i.e., those who did not report “very good” or “excellent” mental health), we were unable to report any findings on engagement or interest in mental health treatment for singles who identified as transgender, or for those who identified as Asian, Native American/American Indian, or as another racial/ethnic group not listed in survey item. Given that the study data stems from a 2022 survey, the exclusion of Asian participants from analyses is particularly noteworthy due to associations observed in the research literature between mental health, race, and the COVID-19 pandemic. For example, Lee and Howard (88) reported increases in the probability of Asian Americans receiving an anxiety or depression diagnosis between 2019 and 2020, and suggest this may be in part due to increased anti-Asian hate crimes stoked by inaccurate media coverage regarding the origins of the COVID-19 virus. As a result, our inability to comment on Asian singles’ engagement in mental health treatment or their interest in attending psychotherapy in the future is an important limitation of this study.

Additionally, the small cell size for Native American/American Indian participants also limited our analysis of their engagement and interest in mental health treatment. Mental health burden is high for this population group. In an analysis of data from the 2012 Behavioral Risk Factor Surveillance System Survey, Asdigian et al. (89) reported that Native American and American Indian adults rated their mental health as “poor” for around 6–7 days out of the past month. Importantly, this study compared the mental health burden of multiracial and single race Native American/American Indian participants and found that multiracial-identified individuals reported worse mental health compared to single-race identified individuals. These findings suggest not only the need for research attention on Native American and American Indian mental health and engagement in treatment, but also intentional targeted recruitment to allow for comparison of multiracial and single race individuals, as over 40% of Native American/American Indian people identify as multiracial (90).

Finally, we lacked information on participants’ education level or area of residency (i.e., urban or rural). Previous research indicates that those with higher levels of education and those living in urban areas are more interested in accessing mental health treatment (34, 91). Future research should take these factors, and those mentioned prior, into account for a more comprehensive understanding of mental health attitudes and preferences.

### Clinical implications and conclusion

Our study on mental health treatment utilization and interest among single adults reveals a complex picture of both high and low levels of engagement with mental health services. Despite a greater propensity among single adults to engage in mental health treatment compared to the general U.S. population, as indicated in Terlizzi and Norris’ (32) study, most participants in our sample were not engaged in mental health treatment at the time of the survey—even though everyone in the final analytic sample self-evaluated their mental health over the last year as being less than “very good” or “excellent”. This underutilization of mental health resources highlights a critical gap in the provision and accessibility of mental healthcare for single adults. Furthermore, our findings reveal demographic disparities in the interest and uptake of mental health treatment based on gender, sexual orientation, and race, pointing to systemic barriers that may influence these trends.

These insights call for a multifaceted approach to address the therapeutic and clinical implications of our findings. Targeted outreach and education are imperative to raise awareness about the benefits of mental health treatment and to reduce stigma, especially in communities with identified disparities in mental health treatment engagement (e.g., Black/African-American and Hispanic/Latino individuals compared to white individuals). Personalized treatment plans that account for the individual’s demographic background and preferences may improve engagement in mental health treatment as well as treatment outcomes.

Moreover, enhancing provider training to better understand and cater to the specific needs and barriers encountered by single adults seeking mental health treatment is crucial. This includes comprehending the impact of systemic discrimination on mental health and implementing strategies to engage underrepresented groups effectively. Collaborating with community organizations can serve as a bridge to reach individuals who are in need of mental health treatment but are currently unengaged, promoting a more inclusive approach to mental healthcare. Finally, these findings underscore the importance of advocacy for policy changes aimed at ensuring equitable access to mental health services for all individuals, regardless of their relationship status, gender, sexual orientation, race, or other factors. By integrating these strategies, we can work towards a more inclusive and effective mental healthcare system that addresses the disparities and underutilization identified in this study, ultimately fostering a supportive environment for the mental well-being of single adults.

## Data availability statement

The data reported in this manuscript were collected as part of a larger data collection. The larger data collection is cross-sectional and has been collected annually since 2010 (except 2018). There are published manuscripts including data from older waves of the survey, but not including the current participants, research topics, or target variables. There are no other manuscripts—published or in preparation—using the data included in the current manuscript. The 2022 wave is the only wave thus far to ask about therapy attendance or interest. Psychiatric medication use has been assessed in prior waves of the survey, but this data has never been reported in any publications. The datasets presented in this study can be found in online repositories. The names of the repository/repositories and accession number(s) can be found in the article/supplementary material.

## Ethics statement

Ethical approval was not required for the studies involving humans because the Singles in America survey is conducted as market research by Match Group; only a de-identified dataset is provided to the researchers. The studies were conducted in accordance with the local legislation and institutional requirements. Written informed consent for participation was not required from the participants or the participants’ legal guardians/next of kin in accordance with the national legislation and institutional requirements because due to the nature of the secondary de-identified data, the present research falls under the category of exempt according to federal regulations at 45 CFRP part 46, paragraph d, number four, item ii (Office for Human Research Protections, 2021).

## Author contributions

AG: Conceptualization, Data curation, Formal analysis, Investigation, Methodology, Project administration, Resources, Software, Supervision, Visualization, Writing – original draft, Writing – review & editing. EK: Formal analysis, Investigation, Project administration, Supervision, Writing – original draft, Writing – review & editing. LW: Conceptualization, Formal analysis, Investigation, Writing – original draft, Writing – review & editing. ZM: Data curation, Formal analysis, Investigation, Software, Validation, Visualization, Writing – original draft, Writing – review & editing. MB-B: Conceptualization, Formal analysis, Investigation, Writing – original draft, Writing – review & editing. OA: Conceptualization, Formal analysis, Investigation, Writing – original draft, Writing – review & editing. JC: Conceptualization, Formal analysis, Investigation, Project administration, Writing – original draft, Writing – review & editing. MP: Conceptualization, Data curation, Investigation, Methodology, Writing – original draft. LB: Data curation, Conceptualization, Validation, Investigation, Writing – original draft. SD: Conceptualization, Formal analysis, Investigation, Writing – original draft. JH: Conceptualization, Investigation, Writing – original draft. JG: Funding acquisition, Methodology, Project administration, Resources, Writing – original draft.
